# Exosomal miRs in Lung Cancer: A Mathematical Model

**DOI:** 10.1371/journal.pone.0167706

**Published:** 2016-12-21

**Authors:** Xiulan Lai, Avner Friedman

**Affiliations:** 1 Institute for Mathematical Sciences, Renmin University of China, Beijing, P. R. China; 2 Mathematical Bioscience Institute & Department of Mathematics, Ohio State University, Columbus, OH, United States of America; Roswell Park Cancer Institute, UNITED STATES

## Abstract

Lung cancer, primarily non-small-cell lung cancer (NSCLC), is the leading cause of cancer deaths in the United States and worldwide. While early detection significantly improves five-year survival, there are no reliable diagnostic tools for early detection. Several exosomal microRNAs (miRs) are overexpressed in NSCLC, and have been suggested as potential biomarkers for early detection. The present paper develops a mathematical model for early stage of NSCLC with emphasis on the role of the three highest overexpressed miRs, namely miR-21, miR-205 and miR-155. Simulations of the model provide quantitative relationships between the tumor volume and the total mass of each of the above miRs in the tumor. Because of the positive correlation between these miRs in the tumor tissue and in the blood, the results of the paper may be viewed as a first step toward establishing a combination of miRs 21, 205, 155 and possibly other miRs as serum biomarkers for early detection of NSCLC.

## Introduction

Lung cancer is the leading cause of cancer-related deaths in the United States and worldwide, and non-small cell lung cancer (NSCLC) constitutes 85% of lung cancer deaths [1, 2]. Five years survival rate for NSCLC is significantly higher for those diagnosed at early stage [3], but there are no reliable tools for early detection of lung cancer. Most lung cancers are first diagnosed on symptoms. Approximately 10% of patients present brain metastasis at the time of initial diagnosis and their mean survival is 4 months [4]. Hence, there is a need for novel noninvasive biomarkers for early lung cancer diagnosis [5].

Exosomes are nano-vesicles of size 30-100 nm in diameter, surrounded by a lipid bilayer, and containing fuctional proteins, mRNAs and microRNAs (miRs). Exosomes are released by various cells, including cancer cells [6]. A growing body of evidence suggests that exosomal miRs may be used as serum biomarkers for prognosis of malignant tumors [5, 7]. Furthermore, exosomal miRs inhibitors have been evaluatedas anti-tumor drugs in experimental and clinical work for several types of cancer, including lung cancer [8, 9].

In the present paper we develop a mathematical model that relates the role of the exosomal miRs in lung cancer tissue to cancer cells proliferation and invasion. Since there is a positive correlation between exosomal miRs in serum and tissue in lung cancer [10–12], the model may serve as a first step toward establishing miRs as reliable serum biomarkers for early detection.

**Fig 1:**
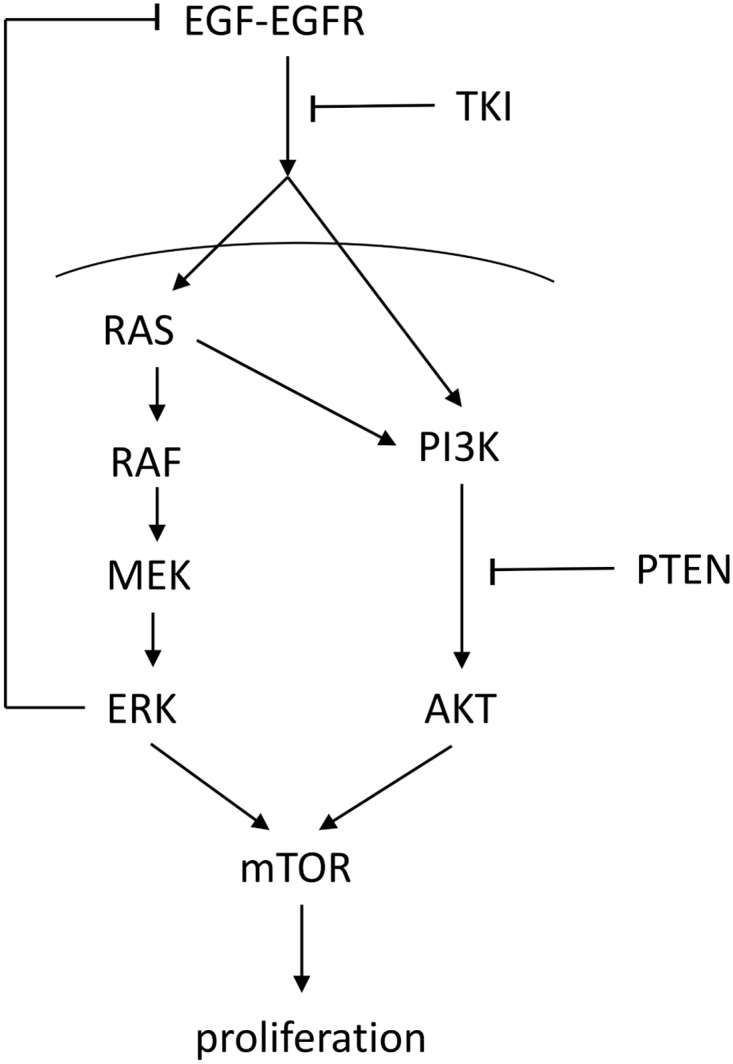
Two pathways, Ras-Raf-MEK-ERK and PI3K-AKT lead to the cell proliferation. Sharp arrows indicate activation/enhancement and blocked arrows indicate inhibition.

A simple schematic of a cell proliferation in the context of cancer is shown in Fig 1. When epidermal growth factor (EGF) ligands to its receptor EGFR, it initiates activation of the Ras-Raf-MEK-ERK pathway [13, 14] and the PI3K-AKT pathway [15–17]. Both pathways lead to cell proliferation [18–21] through activation of mTOR [16, 20, 22]. EGF-EGFR is negatively regulated by ERK [14, 23].

EGFR tyrosine kinase inhibitor (TKI) modulates the activation of both RAS and PI3K, thereby inhibiting the activation of the downstream pathways of EGF-EGFR [13, 24, 25]. PTEN modulates the activation of AKT through converting PIP3, generated by PI3K, to PIP2 by dephosphorylation [15]. When DNA damage occurs, a signaling pathway activates Apaf-1 and caspase 9, forming an apoptosome, which leads to apoptosis through activation of caspase 3 [26, 27].

**Fig 2:**
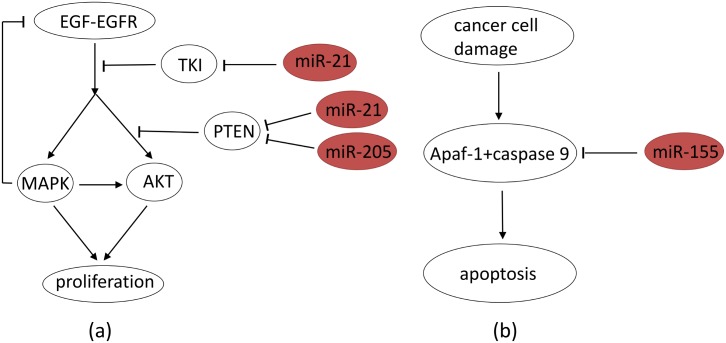
Abbreviated version of Fig 1 depicting the roles of miR-21, miR-205 and miR-155. MAPK represents the Ras-Raf-MEK-ERK signaling pathway and AKT represents the PI3K-AKT signaling pathway. (a) MiR-21 blocks TKI; miR-21 and miR-205 block PTEN. (b) MiR-155 blocks Apaf-1+caspase 9. Sharp arrows indicate activation/enhancement and blocked arrows indicate inhibition.

In NSCLC, the most expressed exosomal miRs are miR-21, miR-155 and miR-205 [10]. In Fig 2(a), we simplified the network of Fig 1 by using MAPK and AKT to represent the Ras-Raf-MEK-ERK and PI3K-AKT pathways. Fig 2(a) also shows the effect of overexpression of miR-21 and miR-205 on NSCLC proliferation. Recent studies established that miR-21 blocks TKI [8, 25], and thus promotes activation of the MAPK and AKT pathways. Also, miR-21 and miR-205 block PTEN [28, 29] and thus promote the activation of the AKT-mTOR pathway. Fig 2(b) shows the effect of overexpression of miR-155. MiR-155 blocks Apaf-1 expression [30] and thus also cellular apoptosis when DNA damage occurs. Hence overexpressions of miR-21 and miR-205 give rise to uncontrolled proliferation, while overexpression of miR-155 leads to reduced apoptosis. MiR-21 and miR-205 have also other targets; in particular it was suggested that miR-21 targets tumor suppressors involved in apopotsis, including Apaf-1, Pdcd4, RhoB and Faslg [31, 32]; hence overexpression of miR-21 reduces apoptosis. However, for simplicity, we focus in this paper on what seems to be the main targets of miR-21 and miR-205 in NSCLC, as shown in Fig 2.

In this paper we consider growth and invasion of lung tumor associated with mutations in EGFR, MAPK and AKT, and its treatment by anti exosomal miRNAs (miR-21, miR-205 and miR-155). We use the mathematical model to determine the efficacy of these drugs under these mutations.

We consider two aspects of tumor progression: (i) Invasion, in which a tumor planar front progresses, in time, away from the main body of the tumor; (ii) Proliferation, in which a small spherical tumor grows in time. In order to focus on the role of the exosomal-miRs, we do not include in the model the immune responses and angiogenesis; thus the model represents an early stage of lung cancer.

## Mathematical model

The mathematical model is based on the network shown in Fig 2. For simplicity we use just one variable, MAPK, to represent the Ras-Raf-MEK-ERK signaling pathway, and AKT to represent the PI3K-AKT signaling pathway. We also combine miR-205 with miR-21 in modeling their effect on blocking PTEN. Table 1 lists the variables used in the mathematical model in unit of g/cm^3^.

**Table 1:** List of variables in unit of g/cm^3^.

Notation	Description
*E*	EGF-EGFR concentration
*M*	active MAPK concentration
*P*_3_	PI3K concentration
*A*	active AKT concentration
*T*	TKI concentration
*P*	PTEN concentration
*Ap*	Apaf-1-caspase 9 apoptosome concnetration
*E*_*C*_	cancer-shed exosome concentration
*m*_1_	exosomal miR-21 concentration (inlcuding miR-205)
$m_{1}^{i}$	cellular miR-21 concentration (inlcuding miR-205)
*m*_2_	exosomal miR-155 concentration
$m_{2}^{i}$	cellular miR-155 concentration
*C*	cancer cells density
*N*	normal healthy cells density

**Equations for proteins** As in [33], the dynamics $\frac{d\vec{y}}{dt}=\vec{f}\left(\vec{y}\right)$ of the proteins within cancer cells will appear in the form

$$\begin{array}{c} \frac{d\vec{y}}{dt}=\vec{f}\left(\vec{y}\right)\frac{C}{C_{0}}, \end{array}$$

where *C*_0_ is the steady state density of cancer cells.

**Equation for EGF-EGFR (*E*).** The equation for EGF-EGFR is given by

$$\begin{array}{c} \frac{dE}{dt}=\left[\lambda_{E}\cdot \frac{1}{1+M/K_{ME}}-d_{E}E\right]\frac{C}{C_{0}}. \end{array}$$ (1)

The coefficient *λ*_*E*_ is the production rate of the EGF-EGFR complex and the factor 1/(1 + *M*/*K*_*ME*_) is the inhibition by ERK [14]; *d*_*E*_ is the degradation rate of *E*.

**Equation for MAPK (*M*).** The MAPK pathway is activated by the EGF-EGFR [13, 14], a process resisted by TKI [8, 13]. Hence

$$\begin{array}{c} \frac{dM}{dt}=\left[\lambda_{M}E\cdot \frac{1}{1+T/K_{TM}}-d_{M}M\right]\frac{C}{C_{0}}, \end{array}$$ (2)

where *d*_*M*_ is the degradation rate of *M*.

**Equation for AKT (*A*).** The activation of the AKT pathway initiates with the activation of PI3K (*P*_3_) by EGF-EGFR directly and also through Ras (which is activated by EGF-EGFR) [15, 16, 19, 20]. In view of the TKI inhibition of EFG-EGFR [24, 25], the equation for *P*_3_ takes the form

$$\begin{array}{c} \frac{dP_{3}}{dt}=\lambda_{P3}E\cdot \frac{1}{1+T/K_{TA}}\cdot \left(1+\lambda_{MA}\frac{S}{K_{SP}+S}\right)-d_{P3}P_{3}, \end{array}$$

where *S* is the concentration of Ras.

AKT is activated by PI3K which is negatively regulated by PTEN [15, 25], so that

$$\begin{array}{c} \frac{dA}{dt}=\lambda_{AP}P_{3}\cdot \frac{1}{1+P/K_{PA}}-d_{A}A. \end{array}$$

We assume that the turnover of PI3K is very fast (the half-life of PI3K is very short [34]) and deduce from the steady state equation of *P*_3_ that

$$\begin{array}{c} P_{3}=\frac{\lambda_{P3}}{d_{P3}}E\cdot \frac{1}{1+T/K_{TA}}\cdot \left(1+\lambda_{MA}\frac{S}{K_{SP}+S}\right). \end{array}$$

Substituting this into the equation for AKT, we obtain

$$\begin{array}{c} \frac{dA}{dt}=\frac{\lambda_{AP}\lambda_{P3}}{d_{P3}}E\cdot \frac{1}{1+P/K_{PA}}\cdot \frac{1}{1+T/K_{TA}}\cdot \left(1+\lambda_{MA}\frac{S}{K_{SP}+S}\right)-d_{A}A. \end{array}$$

Assuming also that the concentration of Ras is proportional to that of MAPK, i.e. *S* = *μM*, we obtain the following equation for AKT:

$$\begin{array}{c} \frac{dA}{dt}=\left[\lambda_{A}E\cdot \frac{1}{1+P/K_{PA}}\cdot \frac{1}{1+T/K_{TA}}\cdot \left(1+\lambda_{MA}\frac{M}{K_{MA}+M}\right)-d_{A}A\right]\frac{C}{C_{0}}, \end{array}$$ (3)

where *λ*_*A*_ = *λ*_*AP*_ *λ*_*P*3_/*d*_*P*3_, *K*_*MA*_ = *K*_*SP*_/*μ*.

**Equation for TKI (*T*).** The production of TKI is inhibited by miR-21 [8, 25]. Recalling that miR-21 is only a fraction of *m*_1_, we write the equation for TKI in the form

$$\begin{array}{c} \frac{dT}{dt}=\left[\lambda_{T}\cdot \frac{1}{1+1/2\cdot \left(m_{1}+m_{1}^{i}\right)/K_{mT}}-d_{T}T\right]\frac{C}{C_{0}}. \end{array}$$ (4)

**Equation for PTEN (*P*).** The expression of PTEN is inhibited by both miR-21 and miR-205 [28, 29]. Hence

$$\begin{array}{c} \frac{dP}{dt}=\left[\lambda_{P}\cdot \frac{1}{1+\left(m_{1}+m_{1}^{i}\right)/K_{mP}}-d_{P}P\right]\frac{C}{C_{0}}. \end{array}$$ (5)

**Equation for Apaf-1-caspase 9 apoptosome (*Ap*).** The expression of Apaf-1 is down-regulated by miR-155 [30]. Hence

$$\begin{array}{c} \frac{dAp}{dt}=\left[\lambda_{Ap}\cdot \frac{1}{1+\left(m_{2}+m_{2}^{i}\right)/K_{m_{2}}}-d_{Ap}Ap\right]\frac{C}{C_{0}}. \end{array}$$ (6)

**Equation for exosome (*E*_*C*_).** Cancer cells shed exosomes at a rate *λ*_*Ec*_*C*. We assume that exosomes are degraded, releasing their miRs, when merging with cancer cells. Taking the rate of this degradation to be $d_{Ec}E_{C}\cdot \frac{C}{K_{C}+C}$, the equation for the concentration of exosomes is given by

$$\begin{array}{c} \frac{\partial E_{C}}{\partial t}-D_{Ec}\Delta E_{C}=\lambda_{Ec}C-d_{Ec}E_{C}\cdot \frac{C}{K_{C}+C}, \end{array}$$ (7)

where the term *D*_*Ec*_Δ*E*_*C*_ represents dispersion (or diffusion) of exosomes.

**Equations for exosomal miR-21 (*m*_1_) and exosomal miR-155 (*m*_2_).** MiR-21 and miR-155 are released from exosomes when exosomes merge with cancer cells. We take the exosomal production rate of miR-21 to be *λ*_*m*_1__*E*_*C*_ ⋅ *C*/(*K*_*C*_ + *C*), and obtain the equation

$$\begin{array}{c} \frac{\partial m_{1}}{\partial t}-D_{m_{1}}\Delta m_{1}=\lambda_{m_{1}}E_{C}\cdot \frac{C}{K_{C}+C}-d_{m_{1}}m_{1}. \end{array}$$ (8)

Similarly, the equation for miR-155 is given by

$$\begin{array}{c} \frac{\partial m_{2}}{\partial t}-D_{m_{2}}\Delta m_{2}=\lambda_{m_{2}}E_{C}\cdot \frac{C}{K_{C}+C}-d_{m_{2}}m_{2}. \end{array}$$ (9)

**Equations for miR-21** ($m_{1}^{i}$) **and miR-155** ($m_{2}^{i}$) **in cancer cells.** Since $m_{1}^{i}$ and $m_{2}^{i}$ lie in cancer cells, they diffuse with same coefficient as cancer cells. Hence, the equations for $m_{1}^{i}$ and $m_{2}^{i}$ are given by

$$\begin{array}{c} \frac{\partial m_{1}^{i}}{\partial t}-D_{C}\Delta m_{1}^{i}=\lambda_{m_{1}^{i}}C-d_{m_{1}}m_{1}^{i}, \end{array}$$ (10)

and

$$\begin{array}{c} \frac{\partial m_{2}^{i}}{\partial t}-D_{C}\Delta m_{2}^{i}=\lambda_{m_{2}^{i}}C-d_{m_{2}}m_{2}^{i}, \end{array}$$ (11)

respectively.

We will apply the mathematical model to consider two phases of tumor progression of lung cancer, invasion and proliferation.

### Model for tumor invasion

**Equation for cancer cell (*C*) in tumor invasion.** The equation for cancer cell is the following:

$$\begin{array}{ccc} \frac{\partial C}{\partial t}-D_{C}\Delta C-\chi \nabla \cdot \left(C\nabla C\right) & = & \left(\lambda_{C1}\frac{M}{K_{M}+M}+\lambda_{C2}\frac{A}{K_{A}+A}\right)\cdot C\left(1-\frac{C}{C_{M}}\right)\\ & & -d_{D}C\cdot \frac{Ap}{K_{Ap}+Ap}-d_{C}C.\;\; \end{array}$$ (12)

Invasion of cancer cells is driven by competition for space and resources [35, 36]. At the early stage of tumor invasion resources are not limited, hence cells undergo migration in the direction of decreased gradient of cancer cells density. On the left-hand side of Eq (12) the term *χ*∇ ⋅ (*C*∇*C*) represents the directed migration of cancer cells in response to the competition for space; *χ* is the ‘directed migration coefficient’. We assume a logistic growth with rate which depends on both MAPK and AKT, since both pathways lead to cell replication; this accounts for the first term on the right-hand side of Eq (12). In addition to natural apoptosis, at rate *d*_*C*_*C*, damage to cancer cells (at rate proportional to *d*_*D*_*C*) leads to apoptosis by formation of the Apaf-1-caspase 9 apoptosome [26, 27]; this is accounted for by the second term on the right-hand side of Eq (12).

**Boundary and initial conditions for tumor invasion model.** We assume that a solid tumor lies in the the half plane *x* < 0, and model the progression of the tumor front in the direction of increasing *x*. We assume that the tumor front is planar, and that it moves in the interval 0 ≤ *x* ≤ 2 from *x* = 0 cm towards the end-point *x* = 2 cm. We impose the following boundary conditions for the cancer cells, exosomes and miRs:

$$\begin{array}{c} \left\{ \begin{array}{cc} E_{C}\left(0,t\right)=E_{C0},\;m_{1}\left(0,t\right)=m_{10},\;m_{2}\left(0,t\right)=m_{20},\;C\left(0,t\right)=C_{0},\;\text{at}\;x=0; & \\ \text{no-flux}\;\text{at}\;x=2. \end{array}\right. \end{array}$$ (13)

Since initially the cancer is confined to *x* < 0, we take zero initial conditions:

$$\begin{array}{c} \left\{ \begin{array}{cc} E(x,0)=M(x,0)=A(x,0)=T(x,0)=P(x,0)=Ap(x,0)=0,\;\text{and} & \\ E_{C}\left(x,0\right)=m_{1}\left(x,0\right)=m_{2}\left(x,0\right)=0,\;C\left(x,0\right)=0,\;\text{for}\;0\leq x\leq 2. \end{array}\right. \end{array}$$ (14)

### Model for tumor proliferation

**Equations for cancer cells (*C*) and normal healthy cells (*N*) in tumor proliferation.** In the tumor invasion model, the directed migration coefficient *χ* represents the directed movement of the invading tumor cells. The precise range of the parameter *χ* is unknown. In order to visualize significant advance of the migrating tumor front we took *χ* to be the ‘relatively’ large. However, in the tumor proliferation model, proliferating cells grow faster than migrating cells [37–39], and the competition for space is primarily a competition with normal healthy cells (with density *N*) [40]. We therefore assume that the directed movement of cells is determined by the condition that the total cell density, *C* + *N*, is constant at each point in the tumor. The term −*χ*∇ ⋅ (*C*∇*C*) in Eq (12) is then neglected and replaced by the term ∇·(uC), where u represents the velocity of cells. The equation for cancer cells is given by

$$\begin{array}{ccc} \frac{\partial C}{\partial t}-D_{C}\Delta C+\nabla \cdot \left(uC\right) & = & \left(\lambda_{C1}\frac{M}{K_{M}+M}+\lambda_{C2}\frac{A}{K_{A}+A}\right)\cdot C\left(1-\frac{C+\varepsilon N}{C_{M}}\right)\\ & & -d_{D}C\cdot \frac{Ap}{K_{Ap}+Ap}-d_{C}C,\;\; \end{array}$$ (15)

where the competition for space with the normal healthy cells is represented by the term *εN* in the logistic growth. We assume that most exosomes shed by cancer cells release their content when they make contact with nearby cancer cells, and therefore keep Eq (7) unchanged. The equation for normal healthy cells, *N*, is given by

$$\begin{array}{c} \frac{\partial N}{\partial t}-D_{N}\Delta N+\nabla \cdot \left(uN\right)=\lambda_{N}N\left(1-\frac{N+\varepsilon C}{C_{M}}\right)-d_{DN}N\cdot \frac{Ap}{K_{Ap}+Ap}-d_{N}N. \end{array}$$ (16)

The competition for space with cancer cells is represented by the term *εC* in the logistic growth term [40].

To simplify the computations, we assume that the tumor is spherical and denote its moving boundary (i.e. its radius) by *r* = *R*(*t*). We also assume that all the densities and concentrations are radially symmetric, that is, functions of (*r*, *t*), where 0 ≤ *r* ≤ *R*(*t*). In particular, $u=u\left(r,t\right)e_{r}$, where $e_{r}$ is the unit radial vector.

**Equation for *u***: We assume that the combined densities of healthy and cancer cells in the tumor is constant (*θ*), and take

$$\begin{array}{c} N+C=\theta =0.6\;\text{g}/{\text{cm}}^{3}. \end{array}$$ (17)

We also assume that *D*_*N*_ = *D*_*C*_. Adding Eqs (16) and (15), we obtain

$$\begin{array}{ccc} \frac{1}{r^{2}}\frac{\partial }{\partial r}\left(r^{2}\theta u\right) & = & [\lambda_{N}N\left(1-\frac{N+\varepsilon C}{C_{M}}\right)\\ & & +\left(\lambda_{C1}\frac{M}{K_{M}+M}+\lambda_{C2}\frac{A}{K_{A}+A}\right)C\left(1-\frac{C+\varepsilon N}{C_{M}}\right)]\\ & & -[\left(d_{DN}N+d_{D}C\right)\cdot \frac{Ap}{K_{Ap}+Ap}+\left(d_{N}N+d_{C}C\right)], \end{array}$$

so that

$$\begin{array}{ccc} u(r,t) & = & \frac{1}{\theta r^{2}}\int_{0}^{r}\xi^{2}[\lambda_{N}N\left(1-\frac{N+\varepsilon C}{C_{M}}\right)\\ & & +\left(\lambda_{C1}\frac{M}{K_{M}+M}+\lambda_{C2}\frac{A}{K_{A}+A}\right)C\left(1-\frac{C+\varepsilon N}{C_{M}}\right)]d\xi \\ & & -\frac{1}{\theta r^{2}}\int_{0}^{r}\xi^{2}\left[\left(d_{DN}N+d_{D}C\right)\cdot \frac{Ap}{K_{Ap}+Ap}+\left(d_{N}N+d_{C}C\right)\right]d\xi . \end{array}$$

We assume that the free boundary *r* = *R*(*t*) moves with the velocity of cells:

$$\begin{array}{c} \frac{dR(t)}{dt}=u\left(R\left(t\right),t\right). \end{array}$$ (18)

**Boundary and initial conditions for tumor proliferation model.** We impose the boundary conditions:

$$\begin{array}{c} \text{No-flux}\;\text{for}\;E_{C}\left(r,t\right),\;m_{1}\left(r,t\right),\;m_{2}\left(r,t\right),\;C\left(r,t\right)\;\text{and}\;N\left(r,t\right)\;\text{at}\;r=R\left(t\right). \end{array}$$ (19)

We assume that the concentrations of proteins TKI, PTEN and Apaf-1 which inhibit tumor growth are ‘relatively’ high, i.e., above the steady state, so that they initially decrease as the tumor begins to increase. We also assume that the remaining proteins which are involved in promoting tumor growth, are initially below their steady state. One choice of initial conditions is given below:

$$\begin{array}{c} \left\{ \begin{array}{cc} R(0)=0.01\text{cm};\\ E\left(r,0\right)=6.7044\times 10^{-4}\text{g}/{\text{cm}}^{3},\;M\left(r,0\right)=7.14\times 10^{-6}\text{g}/{\text{cm}}^{3}, & \\ A\left(r,0\right)=7.396\times 10^{-7}\text{g}/{\text{cm}}^{3},\;T\left(r,0\right)=1.0206\times 10^{-4}\text{g}/{\text{cm}}^{3}, & \\ \;P\left(r,0\right)=2.2748\times 10^{-7}\text{g}/{\text{cm}}^{3},\;Ap\left(r,0\right)=3.4648\times 10^{-5}\text{g}/{\text{cm}}^{3}, & \\ E_{C}\left(r,0\right)=3.6\times 10^{-12}\text{g}/{\text{cm}}^{3},\;m_{1}\left(r,0\right)=2.8\times 10^{-15}\text{g}/{\text{cm}}^{3}, & \\ m_{2}\left(r,0\right)=1.4\times 10^{-15}\text{g}/{\text{cm}}^{3},\;C\left(x,0\right)=0.35\text{g}/{\text{cm}}^{3},\; & \\ N\left(r,0\right)=0.25\text{g}/{\text{cm}}^{3},\;\text{for}\;0\leq r\leq R\left(0\right). \end{array}\right. \end{array}$$ (20)

## Results

### Results for tumor invasion

In simulating the invasion of cancer cells we use the model Eqs (1)–(12), with boundary Conditions (13) and initial Conditions (14), and with the parameters of Tables 2 and 3.

**Table 2:** Summary of parameter values.

Notation	Description	Value used	References
*D*_*Ec*_	diffusion coefficient of exosomes	1.23 × 10^−4^ cm^2^day^−1^	[82] & estimated
*D*_*m*_1__	diffusion coefficient of miR-21	0.13028 cm^2^day^−1^	[61, 83] & estimated
*D*_*m*_2__	diffusion coefficient of miR-155	0.13028 cm^2^day^−1^	[61, 83] & estimated
*D*_*C*_	diffusion coefficient of cancer cells	8.64 × 10^−7^ cm^2^day^−1^	[77]
*D*_*N*_	diffusion coefficient of normal healthy cells	8.64 × 10^−7^ cm^2^day^−1^	[77]
*χ*	directed migration coefficient	3 × 10^−4^ − 3 × 10^−2^ cm^5^g^−1^day^−1^	[33] & estimated
*λ*_*E*_	production rate of EGF-EGFR	1.1741 × 10^−3^ day^−1^ g/cm^3^	[47] & estimated
*λ*_*M*_	production rate of MAPK	1.6499 × 10^−2^ day^−1^	[47, 48] & estimated
*λ*_*A*_	production rate of AKT	2.9422 × 10^−3^ day^−1^	[67, 68] & estimated
*λ*_*MA*_	activation rate of AKT by MAPK (Ras)	1/2	[75] & estimated
*λ*_*T*_	production rate of TKI	8.4013 × 10^−4^ day^−1^ g/cm^3^	estimated
*λ*_*P*_	production rate of PTEN	2.3352 × 10^−4^ day^−1^ g/cm^3^	[47, 70] & estimated
*λ*_*Ap*_	production rate of Apaf-1	4.4095 × 10^−3^ day^−1^ g/cm^3^	[73] & estimated
*λ*_*Ec*_	production rate of exosome by NSCLC cells	9.81 × 10^−9^ day^−1^	[58] & estimated
*λ*_*m*_1__	production rate of miR-21 & miR-205 by *E*_*C*_	0.8626 × 10^−3^ day^−1^	[59, 74] & estimated
*λ*_*m*_2__	production rate of miR-155 by *E*_*C*_	0.4313 × 10^−3^ day^−1^	[59, 74] & estimated
*λ*_*C*1_	growth rate of NSCLC cells due to MAPK	0.6133 day^−1^	[77] & estimated
*λ*_*C*2_	growth rate of NSCLC cells due to AKT	0.3067 day^−1^	[77] & estimated
*λ*_*N*_	growth rate of normal healthy cells	0.092 day^−1^	[77] & estimated
*ε*	competition for space coefficient	0.1	[40]
*d*_*E*_	degradation rate of EGF-EGFR	0.8318 day^−1^	[47] & estimated
*d*_*M*_	degradation rate of MAPK	0.6931 day^−1^	[63–66]
*d*_*A*_	degradation rate of AKT	0.6931 day^−1^	[67, 68]
*d*_*T*_	degradation rate of TKI	0.3466 day^−1^	[69] & estimated
*d*_*P*_	degradation rate of PTEN	22.1807 day^−1^	[70, 71]
*d*_*Ap*_	degradation rate of Apaf-1	2.7726 day^−1^	[73]
*d*_*Ec*_	degradation rate of exosome	21.8 day^−1^	fitted
*d*_*m*_1__	degradation rate of miR-21 and miR-205	0.5545 day^−1^	[74]
*d*_*m*_2__	degradation rate of miR-155	0.5545 day^−1^	[74]
*d*_*C*_	natural death rate of cancer cells	0.023 day^−1^	[77] & estimated
*d*_*D*_	death rate of cancer cells due to DNA damage	0.414 day^−1^	[77] & estimated
*d*_*N*_	natural death rate of normal healthy cells	0.023 day^−1^	[77] & estimated

**Table 3:** Summary of parameter values (continued).

Notation	Description	Value used	References
*E*_0_	S.S. ^1^concentration of EGF-EGFR	7.0573 × 10^−4^ g/cm^3^	[47]
*M*_0_	S.S. concentration of MAPK	8.4 × 10^−6^ g/cm^3^	[48, 50]
*P*_30_	S.S. concentration of PI3K	1.56 × 10^−6^ g/cm^3^	[53, 54]
*A*_0_	S.S. concentration of AKT	9.362 × 10^−7^ g/cm^3^	[53]
*T*_0_	S.S. concentration of TKI	8.5050 × 10^−5^ g/cm^3^	estimated
*P*_0_	S.S. concentration of PTEN	1.88 × 10^−7^ g/cm^3^	[47]
*Ap*_0_	S.S. concentration of Apaf-1-caspase 9	2.84 × 10^−5^ g/cm^3^	[57]
*E*_*C*0_	S.S. concentration of C-shed exosome	3.6 × 10^−10^ g/cm^3^	[58]
*m*_10_	S.S. concentration of miR-21 and miR-205	2.8 × 10^−13^ g/cm^3^	[59, 60]
*m*_20_	S.S. concentration of miR-155	1.4 × 10^−13^ g/cm^3^	[10, 59]
*C*_0_	S.S. density of cancer cell	0.4 g/cm^3^	[62]
*N*_0_	S.S. density of normal healthy cells	0.14 g/cm^3^	[62] & estimated
*C*_*M*_	carrying capacity of NSCLC cells	0.8 g/cm^3^	[62]
*K*_*ME*_	inhibition of EGF-EGFR by ERK	3.936 × 10^−5^ g/cm^3^	[53, 54] & estimated
*K*_*TM*_	inhibition of MAPK by TKI	8.5050 × 10^−5^ g/cm^3^	estimated
*K*_*TA*_	inhibition of AKT by TKI	8.5050 × 10^−5^ g/cm^3^	estimated
*K*_*PA*_	inhibition of AKT by PTEN	1.88 × 10^−7^ g/cm^3^	[47]
*K*_*mT*_	inhibition of TKI by miR-21	0.56 × 10^−13^ g/cm^3^	[59, 60] & estimated
*K*_*mP*_	inhibition of PTEN by miR-21 and miR-205	0.56 × 10^−13^ g/cm^3^	[59, 60] & estimated
*K*_*m*_2__	inhibition of Apaf-1 by miR-155	0.28 × 10^−13^ g/cm^3^	[10, 59] & estimated
*K*_*MA*_	half-saturation of MAPK (Ras) in AKT activation	8.4 × 10^−6^ g/cm^3^	[48, 50] & estimated
*K*_*C*_	half-saturation of cancer cell on merging with exosome	0.4 g/cm^3^	[62] & estimated
*K*_*M*_	half-saturation of MAPK on cancer cell proliferation	8.4 × 10^−6^ g/cm^3^	[48, 50] & estimated
*K*_*A*_	half-saturation of AKT on cancer cell proliferation	9.362 × 10^−7^ g/cm^3^	[47] & estimated
*K*_*Ap*_	half-saturation of Apaf-1 on NSCLC cell apoptosis	2.84 × 10^−5^ g/cm^3^	[57] & estimated

^1^S.S. refers to steady state.

**Fig 3:**
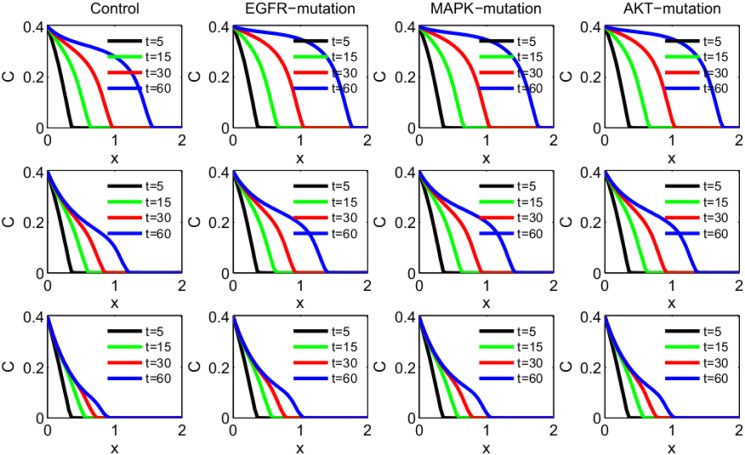
Invasion of cancer cells with density *C*(*x*, *t*). The parameters are as in Tables 2 and 3, and *χ* = 3 × 10^−2^ cm^5^g^−1^day^−1^. (a) The first row: The control case; a mutation in EGFR where *λ*_*E*_ is increased by 1.3-fold; a mutation in MAPK where *λ*_*M*_ is increased by 1.6-fold; a mutation in AKT where *λ*_*E*_ is increased by 1.8-fold. (b) The second row: Using anti-miR-21, where *λ*_*m*_1__ is reduced by a factor 2 compared to the first row. (c) The third row: Using both anti-miR-21 and anti-miR-155, where *λ*_*m*1_ and *λ*_*m*2_ are reduced by a factor 2 compared to the first row. The time is in unit of day, and *x* is in unit of cm.

We explore how specific mutations affect the invasion of the tumor front, and how anti-miR drugs slow the invasion. We consider four cases: (i) the control case (with unspecified mutations), (ii) new mutation in EGFR, (iii) new mutation in MAPK, (iv) new mutation in AKT. In the control case all the parameters are taken to be the same as Tables 2 and 3, and *χ* is taken to be 3 × 10^−2^ cm^5^g^−1^day^−1^. In the case of mutations in EGFR, MAPK or AKT, *χ* is unchanged but the production rates *λ*_*E*_, *λ*_*M*_ and *λ*_*A*_ are increased by some factor. The first row of Fig 3 shows the spatial profile of cancer cell density *C*(*x*, *t*) in the control case, and in the three cases of mutations (in EGFR, MAPK and AKT) at different time points *t* = 5, 15, 30, 60 days. We see that under each of the three mutations the tumor advanced at day 60 by at least 10% more than in the control case. We note however that although the sizes of the invasion under the three mutations are nearly the same, we accounted for the three mutations differently, increasing the production rates of EGFR by a factor 1.3, of MAPK by a factor 1.6 and of AKT by a factor 1.8. The ratios between these factors suggest that a mutation of EGFR increases tumor invasion more than a mutation of MAPK, and a mutation of MAPK increases tumor invasion more than a mutation of AKT. These suggestions, however, need to be verified experimentally.

The second row of Fig 3 shows the effect of anti-miR-21 drug in the control case and in the cases of EGFR, MAPK and AKT mutations. We note that anti-miR-21 reduces the rate of invasion by approximately 17%. When both anti-miR-21 and anti-miR-155 are combined, the reduction is by 40%, as seen in the third row of Fig 3.

**Fig 4:**
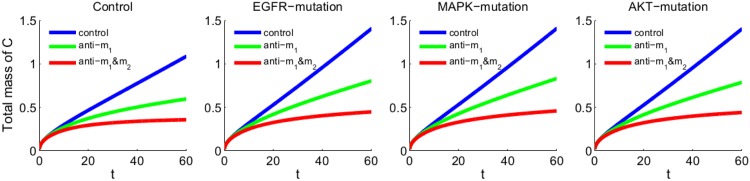
Total linear mass of cancer cells. The mutations and anti-miRs are the same as in Fig 3, and all parameters are the same as in Fig 3. In the legend, anti-*m*_1_ indicates anti-miR-21, and anti-*m*_1_&*m*_2_ indicates the combination of anti-miR-21 and anti-miR-155. The time is in unit of day, and the mass is in unit of g.

Fig 4 simulates the dynamics of the total linear mass of the cancer cells in the control case and the cases of the three mutations when (i) no drug is applied, (ii) anti-miR-21 is applied, and (iii) both anti-miR-21 and anti-miR-155 are applied. We see that, by day 60: (i) each mutation increased the total cancer linear mass by approximately 25% compared to the control case; (ii) anti-miR-21 reduced the total tumor linear mass by approximately 40%; and (iii) in combination with anti-miR-155 the reduction was 65%, and the total linear tumor mass did not grow faster than in the untreated control case.

**Fig 5:**
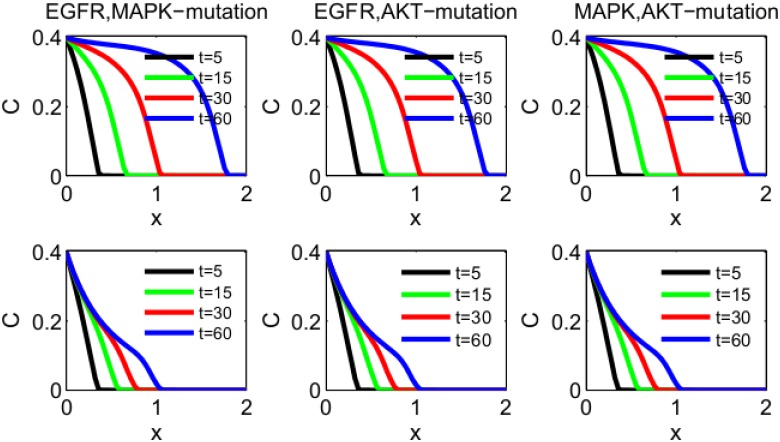
Invasion of cancer cells with density *C*(*x*, *t*). The parameters are as in Tables 2 and 3, and *χ* = 3 × 10^−2^ cm^5^g^−1^day^−1^. (a) Mutations in both EGFR and MAPK, where *λ*_*E*_ and *λ*_*M*_ are increased by 1.15-fold and 1.3-fold, respectively; mutations in both EGFR and AKT, where *λ*_*E*_ and *λ*_*A*_ are increased by 1.15-fold and 1.4-fold, respectively; mutations in both MAPK and AKT, where *λ*_*M*_ and *λ*_*A*_ are increased by 1.3-fold and 1.4-fold, respectively. (b) Using both anti-miR-21 and anti-miR-155, where both *λ*_*m*_1__ and *λ*_*m*_2__ are reduced by a factor 2 compared to the panels in (a). The time is in unit of days, and *x* is in unit of cm.

Similar results can be obtained in the case of multiple mutations. Fig 5 illustrates the case of two mutations (the first row), with reduction in invasion front by approximately 40% at day 60 when the cancer is treated with both anti-miR-21 and anti-miR-155 drugs (the second row).

**Fig 6:**
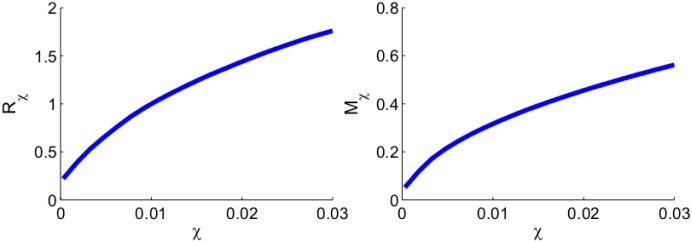
The growth of *R*_*χ*_ and *M*_*χ*_. (a) The distance that the invasion front traveled by day 60 (*R*_*χ*_, in unit of cm). (b) The total linear mass of cancer cells at day 60 (*M*_*χ*_, in unit of g). *χ* ranges from 3 × 10^−4^ to 3 × 10^−2^ cm^5^g^−1^day^−1^. All other parameter values are the same as in the EGFR-mutation case of Fig 3.

Cancer invasion depends on the directed migration coefficient *χ*. In [33] the range of the parameter *χ* was taken to be (3 × 10^−4^, 3 × 10^−2^) cm^5^g^−1^day^−1^. In the simulations of Figs 3–5, we took the largest value *χ* = 3 × 10^−2^ cm^5^g^−1^day^−1^ in order to visualize the invasion of the tumor front over a relatively short period of time. It is reasonable to expect that both tumor invasion and total mass will decrease if *χ* is decreased. This is illustrated in Fig 6 in the case of a tumor with the same EGFR mutation as in Figs 3 and 4. We denote by *R*_*χ*_ the distance traveled by the tumor front by day 60, and by *M*_*χ*_ the total linear mass of the cancer cells by day 60. Fig 6 shows the growth of *R*_*χ*_ and *M*_*χ*_ (at day 60) as *χ* increases from 3 × 10^−4^ to 3 × 10^−2^ cm^5^g^−1^day^−1^: *R*_*χ*_ increases by a factor 8 and *M*_*χ*_ increases by a factor 11 approximately.

**Fig 7:**
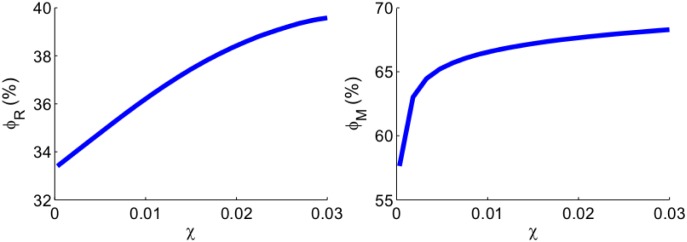
The growth of *ϕ*_*R*_ and *ϕ*_*M*_. (a) The efficacy (*ϕ*_*R*_) of anti-miR drugs in reducing the distance traveled by the tumor front at day 60. (b) The efficacy (*ϕ*_*M*_) of anti-miR drugs in reducing the total linear mass of cancer cells at day 60. *χ* varies from 3 × 10^−4^ to 3 × 10^−2^ cm^5^g^−1^day^−1^. The anti-miR drugs reduce both *λ*_*m*_1__ and *λ*_*m*_2__ by a factor 2. All other parameter values are the same as Fig 6.

We next apply anti-miR-21 and anti-miR-155 drugs to the tumor (by reducing *λ*_*m*_1__ and *λ*_*m*_2__ to *λ*_*m*_1__/2 and *λ*_*m*_2__/2, as in Figs 3 and 4) and denote the corresponding *R*_*χ*_ and *M*_*χ*_ by $R_{\chi }^{m}$ and $M_{\chi }^{m}$. We represent the efficacy of the anti-miRs drugs by $\phi_{R}:=\left(R_{\chi }-R_{\chi }^{m}\right)/R_{\chi }$ and $\phi_{M}:=\left(M_{\chi }-M_{\chi }^{m}\right)/M_{\chi }$, that is, by the percentage of reduction in *R*_*χ*_ and *M*_*χ*_. Fig 7 shows that the efficacy of the drug increases as the directed migration coefficient *χ* increases. The efficacy *ϕ*_*R*_ is approximately 33% when *χ* = 3 × 10^−4^g^−1^day^−1^, and it increases to 40% when *χ* = 3 × 10^−4^g^−1^day^−1^. The efficacy *ϕ*_*M*_ is 57% when *χ* = 3 × 10^−4^g^−1^day^−1^, and it increases to 68% when *χ* = 3 × 10^−4^g^−1^day^−1^.

From Figs 6 and 7 we conclude that as *χ* increases, the tumor invasion and total mass increase, while at the same time the efficacy of anti-miRs drug also increases. The same results (not shown here) hold for other mutations as well as for the control case.

**Fig 8:**
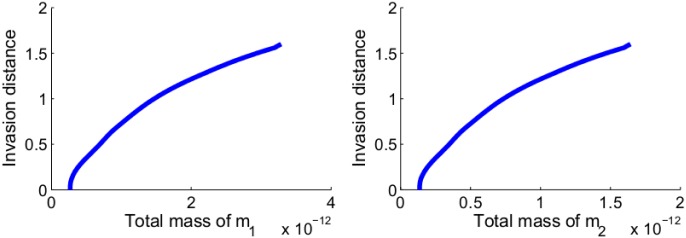
Growth of invasion distance as a function of the total mass of *m*_1_ and total mass of *m*_2_. (a) The invasion distance of cancer cells in the control case as a function of the total mass of miR-21 and miR-205. (b) The invasion distance of cancer cells as a function of the total mass of miR-155. All the parameter values are the same as the control case in Fig 3.

Fig 8 shows the relationship between the invasion distance in the control case to the total mass of miR-21 and the total mass of miR-155 after the first 60 days. Since the concentration of miRs in serum are positively correlated to their concentration in lung cancer tissue [10–12], Fig 8 suggests that miR-21 and miR-155 could potentially be used as serum biomarkers for NSCLC, in line with suggestions made in [5, 7].

### Results for tumor proliferation

The simulations of proliferation of cancer cells are based on the model Eqs (1)–(8), (16)–(18) with boundary Conditions (19) and initial Conditions (20). We increase both *λ*_*C*1_ and *λ*_*C*2_ by a factor 1.4 compared to the values in tumor invasion model in order to account for the fact that proliferating cells grow faster than migrating cells [37–39]. We also increase the steady state *C*_0_ from 0.4 g/cm^3^ to 0.46 g/cm^3^ to reflect the fact that invading cancer cells have sparser density than proliferating cells. We take the steady state density of healthy cells, *N*_0_, to be 0.14 g/cm^3^ so that Eq (17) holds. All the other parameter values are the same as in Tables 2 and 3. We take the initial tumor radius to be *R*(0) = 0.01 cm.

**Fig 9:**
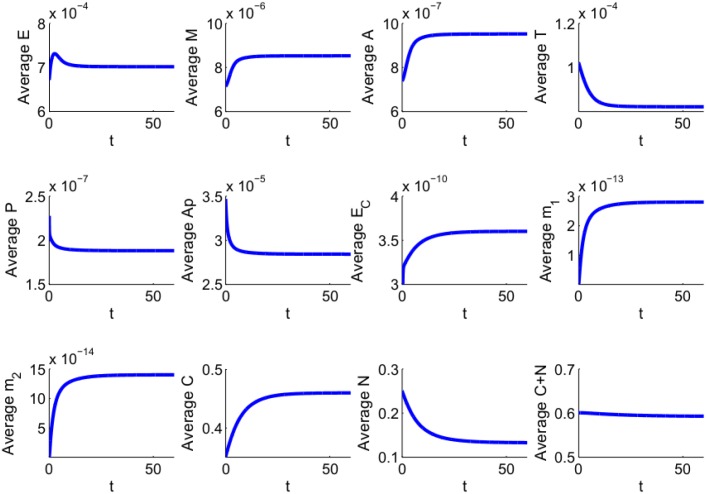
Average densities/concentrations of all the variables in the model. *λ*_*C*1_ and *λ*_*C*2_ are increased by a factor 1.4. *θ* = 0.6 g/cm^3^, *C*_0_ = 0.46 g/cm^3^ and *N*_0_ = 0.14 g/cm^3^. All other parameter values are the same as in Tables 2 and 3.

Fig 9 shows the average concentrations of all the variables over a period of 60 days. Most of the concentrations are either monotone increasing or monotone decreasing in time: the cell growth inhibitors TKI, PTEN and Apaf-1 are decreasing, while the cell growth promoters are increasing. The only exception is the average density of *E*. It is initially increasing since MAPK density is small. But MAPK continues to increase (as *T* keeps decreasing), and after a few days the inhibition by MAPK (or actually ERK, see Fig 1) forces *E* to decrease, and it does so until it reaches a steady state.

We note that in estimating some of the parameters of the model equations we assumed steady-state of the various variables (cells, proteins, miRs). The steady state of the variables in Fig 9 agree approximately with those steady state values, and this establishes consistency of our assumed steady-state values. In particular, the average density of cancer cells stabilize at 0.4631 g/cm^3^, and the average density of normal healthy cells stabilize at 0.1337 g/cm^3^, while *C* + *N* remains approximately equal to 0.6 g/cm^3^ at the entire time.

**Fig 10:**
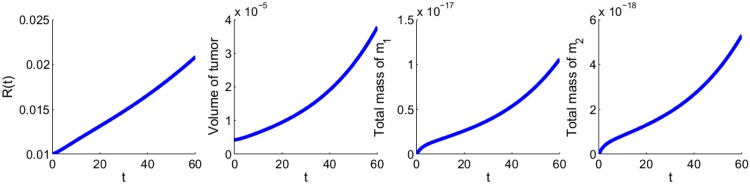
The growth of tumor radius *R*(*t*), tumor volume, total mass of *m*_1_ and total mass of *m*_2_ for the first 60 days. *λ*_*C*1_ and *λ*_*C*2_ are increased by a factor 1.4. *θ* is taken to be 0.6 g/cm^3^. All other parameter values are the same as in Tables 2 and 3.

**Fig 11:**
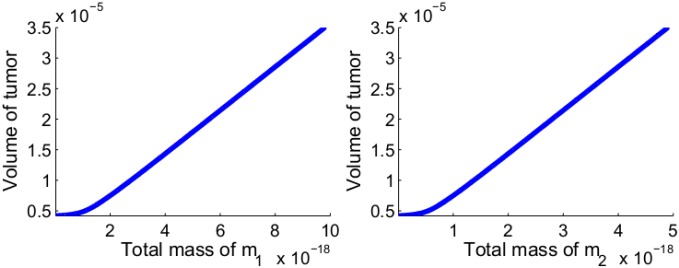
Volume of tumor as a function of the total mass of miR-21 and that of miR-155. (a) The volume of tumor as a function of the total mass of miR-21 after the first 60 days. (b) The volume of tumor as a function of the total mass of miR-155 after the first 60 days. All the parameter values are the same as Fig 10.

Fig 10 shows the growth of the tumor radius and volume, and of the total mass of *m*_1_ and *m*_2_ for the first 60 days. From these profiles we can deduce relations between the total volume of the tumor and the total mass of *m*_1_ and of *m*_2_, at day 60. These relations are shown in Fig 11.

Fig 11 may suggest that miR-21 and miR-155 could be used as biomarkers for determining the volume of NSCLC when this volume is still extremely small; however this suggestion is highly speculative at this time, since other miRs shed from both the cancer cells and immune cells are also circulating in the blood.

We note that the corresponding Fig 8 simulates a different situation, where the tumor is already well established in {*x* < 0} and its front begins to invade into {*x* > 0}.

### Treatment

It is well known that cancer cells in NSCLC lose sensitivity to anti-tumor drugs, for example to paclitaxel, gefitinib and cisplatin, and that some anti-miRs can restore some of this sensitivity. We use our model to explore the effect of anti-miR combined with paclitaxel, gefitinib and cisplatin.

**Fig 12:**
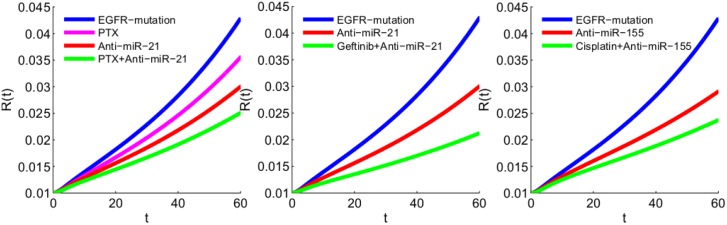
Tumor growth under different drugs. EGFR mutation is accounted for by increasing *λ*_*E*_ by factor 3. (a) Paclitaxel inhibits the division of cells, where *λ*_*Ci*_ are reduced from 1.4*λ*_*Ci*_ to 1.3*λ*_*Ci*_ (*i* = 1, 2); anti-miR-21 is accounted for by reducing *λ*_*m*_1__ to *λ*_*m*_1__/2. (b) Both anti-miR-21 and the gefitinib, a drug that inhibits the EGFR, reduce the growth of tumor; anti-miR-21 is accounted for by reducing *λ*_*m*_1__ to *λ*_*m*_1__/2, and EGFR-inhibitor gefitinib is accounted for by decreasing 3*λ*_*E*_ to 1.5*λ*_*E*_. (c) Anti-miR-155 is accounted for by reducing *λ*_*m*_2__ to *λ*_*m*_2__/1.2 and cisplatin, a drug that induces cancer cell apoptosis, is accounted for by increasing *d*_*C*_ and *d*_*D*_ by factor 1.1.

Paclitaxel drugs (PTX) block progression of mitosis by protecting microtubules against disassembly and preventing chromosomes from achieving metaphase spindle configuration [41]. Researches have observed that paclitaxel-treated cells have defects in mitotic spindle assembly, chromosome segregation and cell division [41]. Experiments *in vivo* by Yung et al. [42] show that anti-miR-21 reduces tumor volume in NSCLC, and the combination of paclitaxel and anti-miR-21 demonstrated greater ability to reduce cancer cell proliferation than either agent administered alone. The simulations in Fig 12(a) mimic this experiment; the effect of PTX is accounted for by reducing 1.4*λ*_*Ci*_ to 1.3*λ*_*Ci*_ (*i* = 1, 2), and the effect of anti-miR-21 is accounted for by reducing *λ*_*m*_1__ to *λ*_*m*_1__/2. We note however that in our model the cancer is at an earlier stage and its volume is much smaller compared to the volume of 0.8 cm^3^ in [42].

Gefitinib is a drug used in the treatment of NSCLC. It blocks the production of EGF-EGFR and thus obstructs the MAPK and AKT pathways [43]. Tumor cells that are initially sensitive to gefitinib may eventually lose sensitivity due to the emergence of acquired resistance [44]. Alternative mechanisms are currently being explored aimed to overcome the development of gefitinib resistance in the patients of NSCLC [44, 45]. Recent studies [8, 25] show that miR-21 modulates gefitinib sensitivity. In particular, Shen et al. [8] demonstrated *in vivo* that reduction in miR-21 significantly restored gefitinib sensitivity by up-regulation of PTEN expression and the inactivation of AKT and MAPK pathways. We can use our model to represent the experimental results of Shen et al. [8]. We account for the effect of gefitinib by reducing 3*λ*_*E*_, in the case of EGFR mutation, to 1.5*λ*_*E*_, and the effect of anti-miR-21 by reducing *λ*_*m*_1__ to *λ*_*m*_1__/2. Fig 12(b) shows that anti-miR-21 alone reduces the growth of tumor volume, but in combination with gefitinib the reduction is significantly larger. This is in qualitative agreement with the results in [8], although here again our model considers an early stage of a tumor whereas, in [8], the tumor volume is already 0.5 cm^3^.

Cisplatin induces cancer cell apoptosis by inhibition of DNA synthesis and repair in cell cycle [46]. The efficacy of cisplatin is initially high, but in the majority of cancer patients it eventually drops due to cisplatin resistance. Many mechanisms of cisplatin resistance have been described, including changes in cellular uptake, drug efflux, increased detoxification of the drug, inhibition of apoptosis, and increased DNA repair [46]. Experiments by Zang et al. [30] show that down-regulation of miR-155 can enhance the sensitivity of lung cancer cells to cisplatin treatment through the induction of DNA damage and apoptosis via the restoration of the mitochondrial apoptotic pathway. The simulation in Fig 12(c) shows that anti-miR-155 alone reduces the tumor volume, but in combination with cisplatin the reduction is significantly higher.

## Discussion and conclusion

Worldwide, lung cancer is the leading cause of cancer deaths, and approximately 85% of lung cancer cases are NSCLC [1, 2]. Five years survival rate for NSCLC is significantly higher for those diagnosed at early stage [3]. Unlike mammography for breast cancer or colonoscopy for colon cancer there are no reliable tools for early detection of lung cancer; most lung cancers are first diagnosed on symptoms. Hence, there is increased focus on identifying biomarkers for detection of NSCLC at early stage [5].

A growing body of evidence suggests that exosomal miRs may be used as serum biomarkers for prognosis of malignant tumors [5, 7]. In NSCLC the highest overexpressed miRs are miR-21, miR-205 and miR-155 [6, 10]. Since exosomal miRs concentration in the blood are positively correlated to their concentrations in tissue [10–12], it is important to understand how the concentrations of miRs 21, 205 and 155 in NSCLC tissue are related to the progression of the cancer, both in terms of tumor growth and tumor-front invasion.

In the present paper we developed a mathematical model that relates the role of the above exosomal miRs in tissue to cancer cells proliferation and invasion. MiRs 21 and 205 regulate cell proliferation through MAPK and PI3K-AKT pathways, while miR-155 regulates apoptosis through the Apaf-1-Caspase 9 complex. The mathematical model includes, separately, invasion and proliferation phases of NSCLC. In invasion, the ‘directed migration coefficient’ *χ* plays a critical role: the tumor front increases as *χ* increases. In order to visualize this monotonic relation, we used a ‘relatively’ large *χ*; *in vivo* this parameter may be much smaller.

In the model of tumor proliferation, what makes the tumor volume grow is the fact that the combined densities of cancer cells (*C*) and healthy cells (*N*) is limited, i.e, *C* + *N* = constant. Simulations of the proliferating model show qualitative agreement with experimental results in the treatment of NSCLC. In these experiments, miR-21 and miR-155 are used to re-establish sensitivity of cancer cells to specific chemotherapeutic drugs.

Simulations of the model of tumor proliferation establish quantitative relations between the total mass of over-expressed miRs (21, 205, 155) and tumor volume. Because of the positive correlation between miRs in cancer tissue and serum [10–12], the present model may be viewed as a first step toward establishing a combination of miRs 21, 205, 155 and possibly additional miRs as serum biomarkers for early detection of NSCLC. As more experimental and clinical data become available, the model could then be refined by estimating more precisely some of the parameters, by expanding the genetic network of Fig 2, and by precisely relating the concentrations of miRs in serum to miRs in lung tissue.

## Parameter values

In the sequal we shall use the following conversion of units: 1Da = 1g/mol, so that

$$\begin{array}{c} 1\;\text{mol}\;\;\;\text{of}\;\;\text{a}\;\;\text{protein}\;\;\text{with}\;\;\text{molecular}\;\;\text{weight}\;m\text{kDa}\;\;\text{has}\;\text{a}\;\;\;\text{mass}\;\;\text{of}\;m\times 10^{3}\text{g}. \end{array}$$ (21)

Also, 1 Molar = 1 mol/L = 10^−3^ mol/cm^3^. Hence 1nM = 10^−12^ mol/cm^3^, and

$$\begin{array}{c} \text{Concentration}\;\;\;\text{of}\;\;\;\text{1nM}\;\;\text{of}\;\;\text{a}\;\;\text{protein}\;\;\text{with}\;\;\text{molecular}\;\;\text{weight}\;m\text{kDa}=m\times 10^{-9}\;\text{g}/{\text{cm}}^{3}. \end{array}$$ (22)

### Steady state concentrations

In some estimating parameters, we use steady state equations; we denote the steady state concentrations of species *X* by *X*_0_.

**EGF-EGFR** Cancer cells express 2 − 3 × 10^6^ EGFR proteins per cell [47]. We take the average to be 2.5 × 10^6^ EGFR per cell, or $\frac{2.5\times 10^{6}}{N_{A}}$ fraction of a mole, per cell, where *N*_*A*_ = 6.022 × 10^23^ is Avogadro’s number. The molecular weight of EGFR is 170kDa [47]. By Eq (21), the mass of EGFR in one cell is $\frac{2.5\times 10^{6}}{N_{A}}\times 170\times 10^{3}\text{g}=7.0573\times 10^{-13}\text{g}$. Assuming that one cell has a volume of 10^−9^ cm^3^, we find that the concentration of EGFR is 7.0573 × 10^−4^ g/cm^3^. We assume that the concentration of EGF-EGFR is not limited by the availability of EGF, hence *E*_0_ = 7.0573 × 10^−4^ g/cm^3^.

**MAPK** The molar concentration of Ras is 0.4*μ*M [48] and its molecular weight is 21kDa [49]. Hence, by Eq (22), the concentration of Ras is *M*_01_ = (0.4 × 10^3^) × 21 × 10^−9^ g/cm^3^ = 8.4 × 10^−6^ g/cm^3^. The molar concentration of Raf is 0.013*μ*M [48, 50], and its molecular weight is 72kDa [48]. Hence the concentration of Raf is *M*_02_ = (0.013 × 10^3^) × 72 × 10^−9^ g/cm^3^ = 0.936 × 10^−6^ g/cm^3^. The molar concentration of MEK is 1.4*μ*M [48, 49] and its molecular weight is 43kDa [51]. Hence the concentration of MEK is *M*_03_ = (1.4 × 10^3^) × 43 × 10^−9^ g/cm^3^ = 6.02 × 10^−5^ g/cm^3^. The molar concentration of ERK is 0.96*μ*M [48, 49] and its molecular weight is 41kDa [52]. Hence the concentration of MEK is *M*_04_ = (0.96 × 10^3^) × 41 × 10^−9^ g/cm^3^ = 3.936 × 10^−5^ g/cm^3^. All the *M*_0*i*_ are of the same order of magnitude. In estimating parameters, we shall use the steady state concentration *M*_0_ of the MAPK. We take it to be that of Ras, that is *M*_0_ = *M*_01_, since steady state of Ras leads to steady state in the MAPK pathway.

**PI3K** The molar concentration of PI3K is 8nM [53, 54], and its molecular weight is 195kDa [55]. Hence, by Eq (22), the concentration of PI3K is *P*_30_ = 8 × 195 × 10^−9^ g/cm^3^ = 1.56 × 10^−6^ g/cm^3^.

**AKT** The molar concentration of AKT is 15.1nM [47], and its molecular weight is 62kDa [47]. Hence, by Eq (22), the concentration of AKT is given by *A*_0_ = 15.1 × 62 × 10^−9^ g/cm^3^ = 9.362 × 10^−7^ g/cm^3^.

**PTEN** The molar concentration of PTEN is 4nM [56], and its molecular weight is 47kDa [56]. Hence, by Eq (22), the concentration of PTEN is *P*_0_ = 4 × 47 × 10^−9^ g/cm^3^ = 1.88 × 10^−7^ g/cm^3^.

**TKI** TKI inhibits the activation of PI3K, and PTEN inhibits the activation of AKT. We assume that these two inhibitions are proportional, that is *T*_0_/*E*_0_ = *P*_0_/*P*_30_. Hence *T*_0_ = *P*_0_*E*_0_/*P*_30_ = 8.5050 × 10^−5^ g/cm^3^.

**Apaf-1** The molar concentration of Apaf-1 ranges from 0.1*μ*M to 0.5*μ*M [57]. We take it to be 0.2*μ*M. The molecular weight of Apaf-1 is 142kDa [57]. Hence, by Eq (22), the concentration of Apaf-1 is (0.2 × 10^3^) × 142 × 10^−9^ g/cm^3^ = 2.84 × 10^−5^ g/cm^3^. We assume that the concentration of Apaf-1-caspase-9 apoptosome is not limited by the availability of caspase 9, hence the concentration is *A*_*p*0_ = 2.84 × 10^−5^ g/cm^3^.

**Exosome** In breast cancer, 10^6^ cancer cells release 5 × 10^8^ exosomes in 24 hours [58]. Assuming that the number of cancer cells in lung cancer is 4 × 10^8^ per cm^3^, and taking the average diameter of exosomes to be 70nm, we estimate the mass density of *E*_*C*_ by *E*_*C*0_ = 3.6 × 10^−10^ g/cm^3^.

**MiR-21, miR-205 and miR-155** For simplicity, we assume steady state of Eqs (10) and (11), so that $m_{1}^{i}=m_{10}^{i},\;m_{2}^{i}=m_{20}^{i}$. We also assume that the cellular concentration of miR-21 is proportional to the exosomal concentration of miR-21, in the sense that $m_{10}^{i}=\gamma m_{10}$, where *m*_10_ is the steady state of *m*_1_. Similarly, we assume $m_{20}^{i}=\gamma m_{20}$, where *m*_20_ is the steady state of *m*_2_. In the simulations, we take *γ* = 10, but the simulations do not change qualitatively if we use different values of *γ* of the same order of magnitude.

The range of molar concentration of miR-21 in healthy individuals is 0.1-0.326 amol/*μ*L [59, 60], and we take it to be 0.2 amol/*μ*L, that is $\frac{0.2\times 10^{-18}}{10^{-3}}\text{mol}/{\text{cm}}^{3}=0.2\times 10^{-3}\text{nM}$. The molecular weight of miR-21 is 7kDa [61]. Hence, by Eq (22), the cellular concentration of miR-21 is (0.2 × 10^−3^) × 7 × 10^−9^ g/cm^3^ = 1.4 × 10^−12^ g/cm^3^. The concentrations of miR-205 and miR-155 are approximately the same as concentration of miR-21 [10]. Since in our model we combine miR-21 and miR-205, we take $m_{20}^{i}=1.4\times 10^{-12}\text{g}/{\text{cm}}^{3}$, while $m_{10}^{i}=2.8\times 10^{-12}\text{g}/{\text{cm}}^{3}$. Hence, *m*_20_ = 1.4 × 10^−13^g/cm^3^ and *m*_10_ = 2.8 × 10^−13^ g/cm^3^.

**NSCLC** We take, for the invasion model, *C*_*M*_ = 0.8 g/cm^3^ and *C*_0_ = 0.4 g/cm^3^ [62].

### Parameter estimation

In the sequal, in expressions of enhancement of the form $\frac{X}{K+X}$ or inhibition of the form $\frac{1}{1+X/K}$, the parameter *K*, the ‘half-saturation’ of *X*, will be taken to be the steady state of *X*. Thus *K*_*MA*_ = *K*_*M*_ = *M*_0_, *K*_*A*_ = *A*_0_, *K*_*Ap*_ = *A*_*p*0_ and *K*_*C*_ = *C*_0_, and *K*_*ME*_ = *M*_0_, *K*_*TM*_ = *K*_*TA*_ = *T*_0_, *K*_*PA*_ = *P*_0_. However, we make an exception in the case of miRs; we assume that the inhibition of protein expressions by miRs is more significant than inhibition by signaling proteins, and take *K*_*mT*_ = *m*_10_/5, *K*_*mP*_ = *m*_10_/5 and *K*_*m*_2__ = *m*_20_/5.

For a species with concentration *X* and half-life *t*_1/2_, the dynamics of its degradation or death is given by

$$\begin{array}{c} \frac{dX}{dt}=-d_{X}X,\;\text{where}\;d_{X}=\text{ln}2/t_{1/2}. \end{array}$$

**Parameter estimation for** Eq (1): The half-life of EGFR ranges from 8 to 24 hours [47]. We take it to be 20 hours, i.e. *t*_1/2_ = 5/6 days, so that *d*_*E*_ = ln2/*t*_1/2_ = 0.8318 days^−1^. From the steady state of Eq (1) with *K*_*ME*_ = *M*_0_, we deduce that *λ*_*E*_ = 2*d*_*E*_*E*_0_ = 1.1741 × 10^−3^ day^−1^ ⋅ g/cm^3^.

**Parameter estimation for** Eq (2): The half-life of KRAS is 12 hours [63]; the half-life of Raf is 30 hours [64]; the half-life of MEK is 8 hours [65], and the half-life of ERK is 24 hours [66]. We take the half-life of the combined pathway MAPK to be 24 hours, that is *t*_1/2_ = 1 day, then *d*_*M*_ = 0.6931 day^−1^. From the the steady state of Eq (2) with *K*_*TM*_ = *T*_0_, we find that *λ*_*M*_ = 2*d*_*M*_*M*_0_/*E*_0_ = 1.6499 × 10^−2^ day^−1^.

**Parameter estimation for** Eq (3): The half-life of AKT ranges from 12 to 36 hours [67, 68]. We take it to be 24 hours, that is, *t*_1/2_ = 1 day, so that *d*_*A*_ = 0.6931 day^−1^. We assume that the activation of AKT pathway by Ras is weaker than the activation by EGF-EGFR, and take *λ*_*MA*_ = 1/2. From the steady state of Eq (3) with *K*_*TA*_ = *T*_0_, *K*_*PA*_ = *P*_0_ and *K*_*MA*_ = *M*_0_, we find that *λ*_*A*_ = (16/5) ⋅ *d*_*A*_*A*_0_/*E*_0_ = 2.9422 × 10^−3^ day^−1^.

**Parameter estimation for** Eq (4): The half-life of the TKI drugs erlotinib, ASD9291 and sunitinib are 36, 50 and 40-60 hours [69]. We take the half-life of TKI to be 48 hours, that is, *t*_1/2_ = 2 days. Hence *d*_*T*_ = 0.3466 day^−1^. From the steady state equation of Eq (4) with *K*_*mT*_ = *m*_10_/5, we find that *λ*_*T*_ = 28.5*d*_*T*_*T*_0_ = 8.4013 × 10^−4^ day^−1^ ⋅ g/cm^3^.

**Parameter estimation for** Eq (5): The half-life of PTEN is 45 minutes [70, 71], that is *t*_1/2_ = 0.03125 days, so that *d*_*P*_ = 22.1807 day^−1^. From the steady state of Eq (5) with *K*_*mP*_ = *m*_10_/5, we find that *λ*_*P*_ = 56*d*_*P*_*P*_0_ = 2.3352 × 10^−4^ day^−1^ ⋅ g/cm^−3^.

**Parameter estimation for** Eq (6): The half-life of Apaf-1 is 1.81h [72]; the half-life of caspase-9 is 6.6h [73]. We assume that Apaf-1-caspase-9 apoptosome is as stable as caspase-9, and take the half-life of the apoptosome to be 6h, that is *t*_1/2_ = 0.25 days. Then *d*_*Ap*_ = 2.7726 day^−1^. From the steady state of Eq (6) with *K*_*m*_2__ = *m*_20_/5, we get *λ*_*Ap*_ = 56*d*_*Ap*_*Ap*_0_ = 4.4095 × 10^−3^ day^−1^ ⋅ g/cm^3^.

**Parameter estimation for** Eq (7): The rate of breakdown of exosomes upon contact with cancer cells is unknown. We take this rate to be *d*_*Ec*_ = 21.8 day^−1^. From the steady state of Eq (7) with *K*_*C*_ = *C*_0_, we get *λ*_*Ec*_ = *d*_*Ec*_*E*_*C*0_/(2*C*_0_) = 9.81 × 10^−9^ day^−1^.

**Parameter estimation for** Eq (8): The half-life of miRs is greater than 24 hours [74]; we take half-life of miR-21 to be 30 hours, i.e. *t*_1/2_ = 1.25 day. Hence *d*_*m*_1__ = 0.5545 day^−1^. From the steady state of Eq (8) with *K*_*C*_ = *C*_0_, we get *λ*_*m*_1__ = 2*d*_*m*_1__*m*_10_/*E*_*C*0_ = 0.8626 × 10^−3^ day^−1^.

**Parameter estimation for** Eq (9): We take the half-life of miR-155 to be 30 hours [74], and then *d*_*m*_2__ = 0.5545 day^−1^. From the steady state of Eq (9) with *K*_*C*_ = *C*_0_, we obtain *λ*_*m*_2__ = 2*d*_*m*_2__*m*_20_/*E*_*C*0_ = 0.4313 × 10^−3^ day^−1^.

**Parameter estimation for** Eq (12): The most common mutations in NSCLC occur in tumor suppressors TP53 and ALK, and in oncogenes PTEN, EGFR, KRAS, LKB1 and BRAF, and mutations seem to occur more frequently in MAPK (KRAS, BRAF) than in PI3K-AKT [21, 75, 76]. We accordingly assume that the proliferation rate of cancer cells through the MAPK pathway is higher than the proliferation rate through the AKT pathway, and take *λ*_*C*1_ = 2*λ*_*C*2_. We also assume that in steady state

$$\begin{array}{c} \frac{1}{2}\lambda_{C1}+\frac{1}{2}\lambda_{C2}=\lambda_{C}, \end{array}$$

and take *λ*_*C*_ = 0.46 day^−1^ [77]. Hence *λ*_*C*1_ = (4/3)*λ*_*C*_ = 0.6133 day^−1^ and *λ*_*C*2_ = (2/3)*λ*_*C*_ = 0.3067 day^−1^.

From the steady state equation of Eq (12) with *K*_*M*_ = *M*_0_, *K*_*A*_ = *A*_0_, *K*_*Ap*_ = *Ap*_0_ and *C*_0_ = *C*_*M*_/2, we get

$$\begin{array}{c} \frac{1}{2}d_{D}+d_{C}=\frac{1}{2}\lambda_{C},\;\text{or}\;d_{D}+2d_{C}=\lambda_{C}. \end{array}$$

We assume that apoptosis rate through intrinsic apoptosis pathway (Apaf-1/Caspace-9) is higher than apoptosis through the extrinsic signaling pathway [78], and take *d*_*D*_ to be larger than *d*_*C*_, so that *d*_*D*_ = 0.9*λ*_*C*_ and 2*d*_*C*_ = 0.1*λ*_*C*_. Hence *d*_*D*_ = 0.414 and *d*_*C*_ = 0.023.

**Diffusion coefficients**: We take *D*_*C*_ = 8.64 × 10^−7^ cm^2^day^−1^ [77]. Diffusion of a sphere is inversely proportional to its diameter. We assume that the average diameter of exosome and cells are 70nm and 10*μ*m respectively. Then we get $D_{E_{C}}=\frac{8.6\times 10^{-7}}{7\times 10^{-3}}=1.23\times 10^{-4}\;{\text{cm}}^{2}\;{\text{day}}^{-1}$. By [79], we have the relation $D_{m_{1}}=\frac{M_{V}^{1/3}}{M_{m_{1}}^{1/3}}D_{V}$, where *D*_*V*_ and *M*_*V*_ are the diffusion coefficient and molecular weight of vascular endothelial growth factor (VEGF), respectively, and *D*_*V*_ = 8.64 × 10^−2^ [77], and *M*_*V*_ = 24*kDa* [80]. The molecular weight of miR-21 is *M*_*m*_1__ = 7 kDa, hence *D*_*m*_1__ = 0.13028 cm^2^day^−1^, and similarly *D*_*m*_2__ = 0.13028 cm^2^day^−1^. The directed migration coefficient *χ* is taken to be in the range 3 × 10^−4^ − 3 × 10^−2^ cm^5^g^−1^day^−1^ [33]. In cell invasion, *χ* should be much larger than in cell growth, so we take *χ* = 2 × 10^−2^ cm^5^g^−1^day^−1^ for the model of tumor invasion.

**Parameter estimation for** Eq (16): Cancer cells can survive in hostile environment better than normal cells, so the apoptosis rate *d*_*N*_ should be somewhat larger than *d*_*C*_; we take *d*_*N*_ = 1.1*d*_*C*_ = 0.0253 day^−1^ and *λ*_*N*_ = 0.8*λ*_*C*_ = 0.368 day^−1^. Since cancer cells replication is less susceptible to damage, *d*_*DN*_ should be larger than *d*_*D*_; we take *d*_*DN*_ = 1.1*d*_*D*_ = 0.4554 day^−1^. We choose *ε* = 0.1, as in [40]. We assume that *C*_0_ in the proliferation phase to be somewhat larger than the average density 0.4 g/cm^3^ in the invasion phase; we take *C*_0_ = 0.46 g/cm^3^ and *N*_0_ = 0.14 g/cm^3^.

## Sensitivity analysis

**Fig 13:**
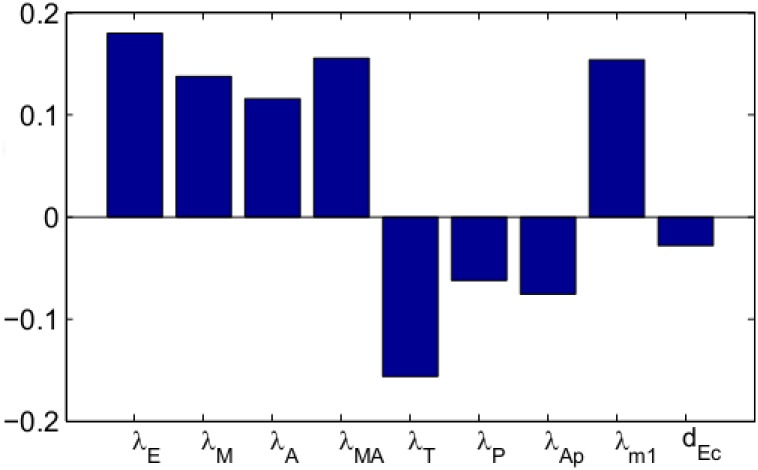
Statistically significant PRCC values (p-value < 0.01) for *R*(*t*) at day 60.

We performed sensitivity analysis on some of the production parameters of the system Eqs (1)–(9), (15)–(18); we also included the important parameter *d*_*Ec*_ which was only fitted. Following the method of [81] we performed Latin hypercube sampling and generated 1000 samples to calculate the partial rank correlation coefficients (PRCC) and the p-values with respect to the tumor radius at day 60. We have taken the range of each parameter from 1/2 to twice its value in Table 2. The results are shown in Fig 13.

We see that the production rates that increase proliferation through EGF-EGFR → MAPK and EGF-EGFR → AKT pathways, namely, (*λ*_*E*_, *λ*_*M*_) and (*λ*_*E*_, *λ*_*M*_, *λ*_*MA*_) are positively correlated to tumor radius. On the other hand the production rates of cell-replication inhibitors, *λ*_*T*_ and *λ*_*P*_, and the production rate of apoptosis-promotor apoptosome, *λ*_*Ap*_, are negatively correlated. Since miR-21 blocks the inhibitors *T* and *P*, so if *λ*_*m*_1__*E*_*C*_ grows the tumor volume will increase. Hence *λ*_*m*_1__ is positively correlated and *d*_*Ec*_ is negatively correlated.

## Computational method

We employ moving mesh method to numerically solve the free boundary problem for the tumor proliferation model. To illustrate this method, we take Eq (16) as example and rewrite it as the following form:

$$\begin{array}{c} \frac{\partial N(r,t)}{\partial t}=D_{N}\Delta N\left(r,t\right)-div\left(uN\right)+F,\; \end{array}$$ (23)

where *F* represents the term in the right hand side of Eq (16). Let $r_{i}^{k}$ and $N_{i}^{k}$ denote numerical approximations of i-th grid point and $N(r_{i}^{k},n\tau )$, respectively, where *τ* is the size of time-step. The discretization of Eq (23) is derived by the fully implicit finite difference scheme:

$$\begin{array}{c} \frac{N_{i}^{k+1}-N_{i}^{k}}{\tau }=D_{N}\left(N_{rr}+\frac{N_{r}}{r_{i}^{k}}\right)-\left(\frac{u_{r}}{r_{i}^{k+1}}+u_{i}^{k+1}\right)N_{i}^{k+1}+F_{i}^{k+1}, \end{array}$$ (24)

where $N_{r}=\frac{h_{-1}^{2}N_{i+1}^{k+1}-h_{1}^{2}N_{i-1}^{k+1}-\left(h_{1}^{2}-h_{-1}^{2}\right)N_{i}^{k+1}}{h_{1}\left(h_{-1}^{2}-h_{1}h_{-1}\right)}$, $N_{rr}=2\frac{h_{-1}N_{i+1}^{k+1}-h_{1}N_{i-1}^{k+1}+\left(h_{1}-h_{-1}\right)N_{i}^{k+1}}{h_{1}(h_{1}h_{-1}-h_{-1}^{2}})$, $u_{r}=\frac{h_{-1}^{2}u_{i+1}^{k+1}-h_{1}^{2}u_{i-1}^{k+1}-\left(h_{1}^{2}-h_{-1}^{2}\right)u_{i}^{k+1}}{h_{1}\left(h_{-1}^{2}-h_{1}h_{-1}\right)}$, $h_{-1}=r_{i-1}^{k+1}-r_{i}^{k+1}$ and $h_{1}=r_{i+1}^{k+1}-r_{i}^{k+1}$. The mesh moves by $r_{i}^{k+1}=r_{i}^{k}+u_{i}^{k+1}\tau$, where $u_{i}^{k+1}$ is solved by the velocity equation.
